# A Pathogenic *ANK1* c.5096G>A Mutation Disrupting the Ankyrin‐1/*β*‐Spectrin Interaction in Hereditary Spherocytosis

**DOI:** 10.1155/humu/7320235

**Published:** 2026-08-19

**Authors:** Xu Linglong, Gao Jingwen, Zhu Wenting, Lu Nina, Ding Xiaoxiao, Zhao Lei, Zhou Siyun, Cai Zihao, Zhong Zhengyang, Zhu Bo, Chen Sai, Guo Yanrong, Chen Jianlin, Chen Jie, Ding Xiaofei, Chen Guang

**Affiliations:** ^1^ Department of Hematology, Taizhou Central Hospital (Taizhou University Hospital), School of Medicine, Taizhou University, Taizhou, Zhejiang, China, tzc.edu.cn; ^2^ Zhejiang Key Laboratory of New Drug Development for Central Nervous System Diseases, Taizhou University, Taizhou, Zhejiang, China, tzc.edu.cn; ^3^ Department of Pharmacology, School of Medicine, Taizhou University, Taizhou, Zhejiang, China, tzc.edu.cn; ^4^ Department of Laboratory Medicine, Taizhou Central Hospital (Taizhou University Hospital), School of Medicine, Taizhou University, Taizhou, Zhejiang, China, tzc.edu.cn

**Keywords:** *β*-spectrin, *ANK1* c.5096G>A mutation, ankyrin-1 (ANK1), hereditary spherocytosis (HS)

## Abstract

Hereditary spherocytosis (HS) is a common inherited hemolytic anemia characterized by spherical erythrocytes, splenomegaly, and increased osmotic fragility, with *ANK1* mutations being the most frequent cause. We identified a novel *ANK1* mutation (c.5096G>A, p.R1699K) in a patient with classic HS phenotypes and a family history of hemolytic anemia. To explore its pathogenicity, we performed a series of hematological and morphological analyses. Compared with wild‐type mice, the *Ank1* c.5198G>A knock‐in mice displayed typical HS features. Hematologically, they showed reduced MCV, MCH, and MCHC. The EMA fluorescence intensity was significantly decreased, and RBCs exhibited increased osmotic fragility. Morphologically, peripheral smears revealed abundant spherocytes, and electron microscopy showed a transformation from normal biconcave discs to spherical erythrocytes. Additionally, the *Ank1* c.5198G>A knock‐in mice presented with significant splenomegaly. Mechanistically, coimmunoprecipitation analysis indicated a significantly weakened association between ankyrin‐1 and *β*‐spectrin in the *Ank1* c.5198G>A knock‐in mice. This study provides in vivo evidence that the Ank1 R1733K mutation impairs erythrocyte membrane stability and cytoskeletal integrity, recapitulating the HS phenotype in mice. Our findings support the pathogenicity of the *ANK1* c.5096G>A mutation and offer a useful model for further investigating the molecular mechanisms of HS.

**Novelty Statement**

1. We identified a novel ANK1 mutation (c.5096G>A, p.R1699K) in a patient with classic hereditary spherocytosis (HS) phenotypes and a family history of hemolytic anemia
2. Our work provides functional evidence supporting the pathogenicity of the ANK1 c.5096G>A mutation in animal model and the patient.
3. Our work offers a useful model for further investigating the molecular mechanisms of HS.

## 1. Introduction

HS is a common inherited hemolytic anemia, characterized by spherocytes that are osmotically fragile and are prematurely destroyed in the spleen [1]. It arises from mutations in genes encoding key erythrocyte membrane proteins (ANK1, SPTB, SPTA1, EPB42, and SLC4A1) that maintain RBC membrane integrity [2, 3]. Among these, ANK1 mutations are most prevalent (40%–65% of HS cases); they disrupt the ankyrin–spectrin–band 3 network, causing membrane instability, increased fragility, and hemolysis [4, 5].

The *ANK1* gene (chromosome 8p11.21) contains 42 exons and encodes the 210‐kDa ankyrin‐R, a central scaffold of the erythrocyte membrane skeleton [4]. Its function relies on three domains: an N‐terminal membrane‐binding domain (24 ankyrin repeats, binds band 3/RhAG), a central Spectrin‐binding domain (ZU5‐UPA‐ZU5 modules, anchors *β*‐spectrin dimers), and a C‐terminal regulatory domain (governs protein stability). During erythropoiesis, ANK1 mediates vertical integration of the membrane skeleton: Its N‐terminus tethers band 3 tetramers, whereas the central domain links the *β*‐spectrin–actin horizontal network, forming a “sandwich” structure that ensures RBC biconcavity and deformability. Physiologically, ANK1 maintains membrane integrity via lipid bilayer‐cytoskeleton coupling (preventing vesiculation) and stabilizes surface antigens; it also modulates RBC metabolism by interacting with pyruvate kinase (PKLR) to regulate glycolysis for ATP‐dependent membrane stabilization [5]. ANK1 deficiency disrupts vertical linkages, causing progressive membrane loss and morphological abnormalities.

*ANK1* mutations cause 40%–50% of HS cases, with > 200 pathogenic variants reported. Major types include nonsense (30%), frameshift (25%), splice‐site (20%), missense (15%), and large deletions (10%), with hotspots in Exon 8 (ankyrin repeats), Exons 13–20 (Spectrin‐binding domain), and Exon 37 (regulatory domain). Nonsense/frameshift mutations (e.g., c.648G>A/p.Trp216 ^∗^, c.3614_3617delCTTG/p.Ser1205Tyrfs ^∗^31) produce truncated proteins with dominant‐negative effects; splice‐site mutations (e.g., Intron 29 c.4570‐1G>A) cause exon skipping; missense mutations (e.g., Exon 13 c.1424T>C/p.Leu475Pro) reduce spectrin affinity; promoter deletions decrease ANK1 expression by > 50%. These defects uncouple the cytoskeleton from the lipid bilayer, causing membrane microvesiculation, surface area loss, and rigid spherocytes—these cells undergo splenic sequestration, leading to chronic extravascular hemolysis [4].

In this study, we identified an *ANK1* c.5096G>A mutation in a clinical HS patient and investigated its pathogenic role. Using CRISPR/Cas9, we introduced the homologous *Ank1* c.5198G>A mutation into C57BL/6J mice to establish a knock‐in model. We aim to determine if this mutation alone induces spherocytosis, membrane instability, and hemolytic anemia, providing the first functional validation of ANK1 c.5096G>A in HS pathogenesis. Findings will enhance understanding of HS molecular mechanisms and inform refined genetic diagnosis and targeted therapies.

## 2. Materials and Methods

### 2.1. Participants

Participants gave informed consent for this study, which was approved by the Institutional Review Board of Taizhou Central Hospital (Approval No. 2024‐07‐39; Taizhou, China).

### 2.2. Genetic Analysis

Peripheral blood (3 mL) was collected from a female HS patient and her family. Genomic DNA (gDNA) was extracted from peripheral blood leukocytes using a MagBead‐based kit (Tiangen Biotech, Beijing); its concentration was measured with a Qubit 3.0 fluorometer (Invitrogen, United States) and integrity verified via 1% agarose gel electrophoresis.

Qualified gDNA was sheared using the VAHTS Universal DNA Library Kit (Vazyme, Nanjing), followed by liquid‐phase capture with the xGen Exome Research Panel v2.0 (Integrated DNA Technologies, United States) to construct a whole‐exome library. The library was sequenced on the MGISEQ‐T7 platform (BGI Genomics) for 150‐bp paired‐end whole‐exome sequencing (WES) (experiments conducted by Wuhan Primbio Medical Laboratory).

Bioinformatics analysis showed the patient′s sample generated > 10 Gb data, with > 99% of regions having > 20× coverage and average depth > 12×. Reads were aligned to the GRCh37/hg19 genome using BWA (v0.7.16a); low‐quality reads/adapters and PCR duplicates were removed with fastp (v0.21.0) and sambamba (v0.8.0), respectively. Variant detection (SNVs, small indels < 50 bp) was performed on BAM files via GATK (v4.0.8.1). Variants were filtered by depth (> 20×) and quality (QualByDepth > 2), then annotated with ANNOVAR (v20200608). Further filtering prioritized minor allele frequency, protein effects, in silico scores, disease genetic patterns, and clinical phenotype consistency. Recessive splicing sites were predicted using HSF (v3.1), varSEAK, and SpliceAI. Candidate variant pathogenicity was interpreted per ACMG/AMP and ClinGen SVI guidelines, and validated by Sanger sequencing.

### 2.3. Generation of *Ank1* c.5198G>A Knock‐In Mice

C57BL/6J mice were obtained from the Shanghai Model Organisms Center. All animal procedures complied with regulations of the Taizhou University Medical School Animal Center and the Institutional Animal Care and Use Committee (TZXY‐2023‐20231095). Mice were housed in a specific pathogen‐free facility under a 12‐h light/12‐h dark cycle, with free access to food and water.

**Figure 1:**
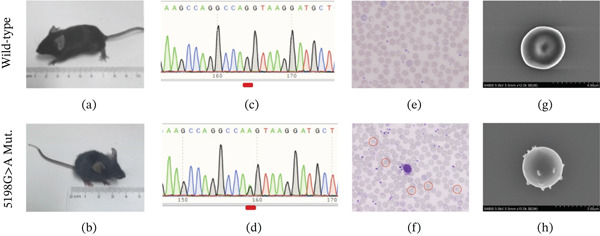
Generation and morphological characterization of *Ank1* c.5198G>A (p. R1733K) knock‐in mice. (a, b) Representative whole‐body images of (a) wild‐type and (b) *Ank1* c.5198G>A knock‐in mice. (c, d) Sanger sequencing confirmation of *Ank1* c.5198G>A knock‐in mice. (c) Wild‐type sequence; (d) heterozygous knock‐in sequence showing the G > A transition (red line). (e, f) Wright–Giemsa–stained peripheral blood smears. (e) Wild‐type red blood cells (RBCs) display normal biconcave disc morphology with central pallor. (f) Mutant RBCs show abundant spherocytes (round, no central pallor, red circle). (g, h) Scanning electron microscopy (SEM) images of RBCs. (g) Wild‐type RBCs retain biconcave shape. (h) Mutant RBCs exhibit spherical morphology with loss of biconcavity.

Heterozygous *Ank1* c.5198G>A (p.R1733K) knock‐in mice (targeting murine Ank1, ENSMUSG00000031543) were generated by Shanghai Model Organisms (Shanghai, China). The targeting guide RNA sequence was 5 ^′^‐ACCCACCGAGCATCCTTACC‐3 ^′^. In vitro transcribed Cas9 mRNA, chimeric sgRNA, and a 241‐bp single‐stranded oligodeoxynucleotide (ssODN) were injected into C57BL/6 mouse zygotes (Figure 1a). F0 mice were validated by sequencing using primers: m*Ank1*‐F (5 ^′^‐CAGATGCCACAAATGGCCTG‐3 ^′^) and m*Ank1*‐R (5 ^′^‐ACGGTACCATGTCCCAAACC‐3 ^′^). Mice with the expected single‐nucleotide mutation were crossed with wild‐type (WT, +/+) C57BL/6 mice to generate F1 knock‐in mice. The ssODN sequence was: 5 ^′^‐TCTCAGGCACAGAGCAGGACACGGAGACTGAAGTGTCTCTTGTTTCAGGCCAGCAGCGAGTTCACGCCCGAATCACAGACTCACCCTCAGTGAGGCAGGTGCTGGACAGAAGCCAGGCCAAGTAAGGATGCTCGGTGGGTCTCTTCTGTCTTCCTGGACTTTGGGGCCAGCTATCCTGGCACTGGGCGCAGGCAGAAGGTTTGGATGCTGCTCTTCATCCTGCATTGTAAACACGAATGCC‐3 ^′^.

### 2.4. Hematological Analysis

To assess hematological parameters, whole blood was collected via retro‐orbital bleeding and analyzed using an automated hematology analyzer by following the instrument manual. Reticulocyte counts were determined by flow cytometry using thiazole orange staining (BD Biosciences).

### 2.5. Eosin‐5 ^′^‐Maleimide (EMA) Flow Cytometry

Freshly isolated RBCs (5 *μ*L) were incubated in 200 *μ*L of EMA dye solution for 1 h in the dark at room temperature. After incubation, cells were washed three times with PBS and resuspended in 500‐*μ*L PBS for flow cytometric analysis. Fluorescence intensity (mean fluorescence intensity [MFI]) was measured using a BD FACSCalibur flow cytometer.

### 2.6. Osmotic Fragility Assay

RBCs from WT and *Ank1* c.5198G>A mutation (R1733K) mice were incubated in NaCl solutions ranging from 0.7% to 0.3% for 30 min at room temperature. After centrifugation, the supernatant absorbance was measured at 540 nm. Hemolysis (%) was calculated relative to complete lysis (100%) in distilled water.

### 2.7. Peripheral Blood Smears and Scanning Electron Microscopy (SEM)

Thin blood smears were prepared from the EDTA‐treated blood samples and stained using the Wright–Giemsa staining method with methanol, thiazine, and eosin dyes (Baso Diagnostics Inc., Zhuhai, China). Olympus BX35 microscope (Tokyo, Japan) with LABB C1C22 lens was used to take photographs (Beion v 4.20). Smears were examined under light microscopy at 1000× magnification.

For SEM imaging of RBC morphology, freshly isolated RBCs were fixed with 2.5% glutaraldehyde, dehydrated in ethanol, and coated with gold‐palladium. Imaging was performed using a scanning electron microscope at 5‐kV acceleration voltage.

### 2.8. Coimmunoprecipitation (co‐IP) Assay

Red blood cells (RBCs) were lysed in ice‐cold RIPA buffer (Beyotime Biotechnology, Haimen) with protease/phosphatase inhibitors (Yeasen, Shanghai), after which lysates were clarified by centrifugation (12,000 g, 15 min, 4°C). Protein concentration was measured via BCA kit (Pierce), and for each sample, 500‐*μ*g total protein was incubated overnight at 4°C with 2‐*μ*g anti–ankyrin‐1 antibody (Santa Cruz, sc‐376187) or isotype IgG, then with 30‐*μ*L protein A/G magnetic beads (Beyotime, Haimen) overnight at 4°C with gentle rotation. The beads were washed three times with lysis buffer to remove nonspecific proteins, and bound proteins were eluted by boiling in SDS loading buffer, run on SDS‐PAGE, and immunoblotted with antispectrin antibody (Abcam, ab129065). Finally, blots were developed with ECL reagents (Biosharp, BL523B), and images captured using GelView 6000 Plus (Guangzhou Biolight). Densitometric analysis was performed using ImageJ software (NIH) on three independent co‐IP experiments. The relative band intensity of *β*‐spectrin coprecipitated with ankyrin‐1 was normalized to the input control and compared between mutant and WT mice.

### 2.9. Statistical Analysis

All data were expressed as mean ± standard deviation (SD). Statistical significance was determined using, Welch′s *t*‐test for normally distributed data, Mann–Whitney *U* test for nonnormally distributed data. *p* < 0.05 was considered statistically significant. All analyses were performed using GraphPad Prism 10. Sample sizes for each experiment were determined based on previous studies and availability. For mouse experiments, *n* ≥ 3 biological replicates were used for each group, consistent with standard practices for hematological phenotyping. Specific sample sizes for each assay are indicated in the corresponding figure legends or tables.

## 3. Results

### 3.1. Clinical and Genetic Findings of the Proband and Her Family Members

A 9‐year‐old female proband (born in 2014) was admitted for persistent jaundice, darkened urine, anorexia, and nausea. Physical examination showed mild jaundice and splenomegaly (spleen palpable 1 cm below costal margin); no other abnormalities were found.

Complete blood count (CBC) results: white blood cell (WBC) count 5.9 × 10^9^/L, RBC count 4.09 × 10^12^/L, hemoglobin 118 g/L, platelet (PLT) count 389 × 10^9^/L, and reticulocyte count 21.50 × 10^10^/L (5.69%, reference 0.50%–2.80%), with a negative Coombs test. Liver function tests revealed elevated total bilirubin (115.5 *μ*mol/L), with direct bilirubin 45.3 *μ*mol/L and indirect bilirubin 70.2 *μ*mol/L. Hemolysis‐related tests showed increased RBC osmotic fragility and reduced MFI in the erythrocyte EMA binding assay (Table 1).

**Table 1:** Laboratory detects of hemolysis and erythrocyte membrane stability in the proband.

Assay name	Result	Reference range
1. Hemoglobin analysis		
Antialkaline hemoglobin	1.10%	0%–2.5%
Hemoglobin A_2_	3.00%	2.5%–3.5%
Isopropanol test	Negative	Negative
Heinz body test	2%	0%–1%
Hemoglobin H inclusion test	0%	0%–5%
2. Erythrocyte metabolic enzymes activity		
Pyrimidine 5 ^′^‐nucleotidase	3.25	2.60–3.52
Glucose‐6‐phosphate dehydrogenase	1544 U/L	N/A
Pyruvate kinase	Normal	Normal
Glucose phosphate isomerase	Normal	Normal
3. Erythrocyte membrane stability assay		
Acidified glycerol lysis	25 s	> 290 s
Initial hemolysis in osmotic fragility	0.60%	0.44%–0.48%
Complete hemolysis in osmotic fragility	0.40%	0.28%–0.36%
Hyperosmotic sucrose lysis	8.40%	0%–16.9%
Red cell incubation osmotic fragility	0.57%	0.44%–0.60%
4. EMA flow cytometry analysis		
Reduction of MFI in erythrocyte E5 ^′^M	23.89%	< 16%
5. Reticulocyte and direct antiglobulin test		
Reticulocyte percentage	5.69%	0.50%–2.80%
Reticulocyte count	21.50 × 10^10^/L	2.40–14.00 × 10^10^/L
Spherocytes percentage	10%	/
Direct Coombs test	Negative	Negative

Abbreviations: EMA, eosin‐5 ^′^‐maleimide; MFI, mean fluorescence intensity.

**Figure 2:**
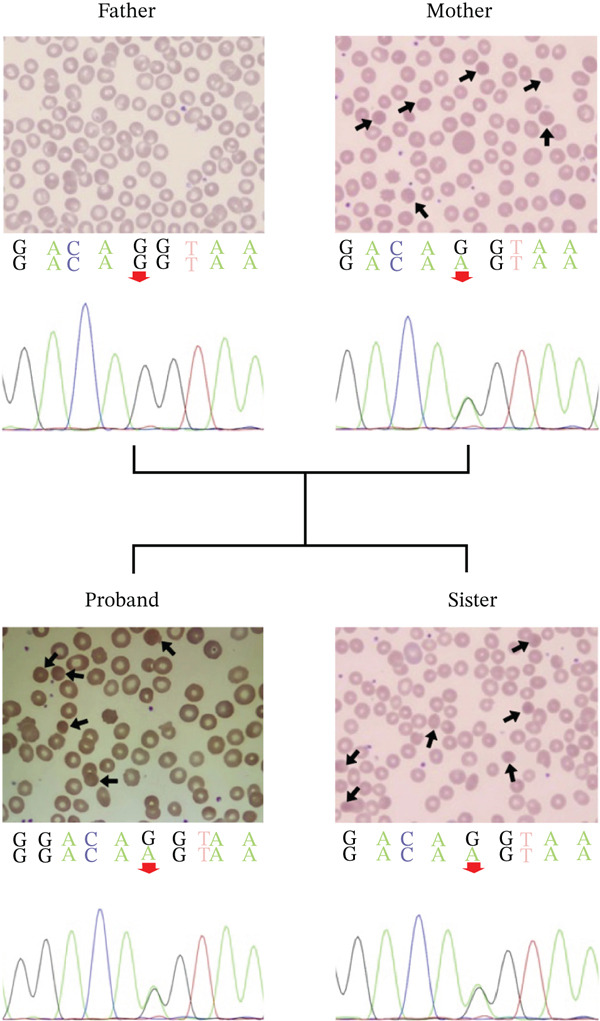
Peripheral blood smears and Sanger sequencing of an HS‐affected family carrying the *ANK1* c.5096G>A (p.R1699K) mutation. Top panels: Wright–Giemsa–stained peripheral blood smears from the father (unaffected), mother, proband, and elder sister. Spherocytes (black arrows) are visible in the mother, proband, and sister, but not in the father. *S* *c* *a* *l* *e* *b* *a* *r* = 10 *μ* *m*. Bottom panels: Sanger sequencing chromatograms confirming the *ANK1* c.5096G>A mutation (red arrow) in the proband, mother, and sister; the father shows the wild‐type sequence.

The proband had a positive family history of inherited hematologic disorders: Her maternal grandmother was diagnosed with HS, her mother had splenomegaly and a splenectomy history, and her elder sister also had splenomegaly (Table 2). Combined with clinical and laboratory findings, HS was suspected. Peripheral blood smear and WES were performed for the proband and her family (Figure 2). Peripheral blood smears showed normal RBC morphology in the father, whereas spherocytes were observed in the mother, proband, and elder sister. Sanger sequencing identified a novel *ANK1* variant (c.5096G>A, p.R1699K) in the proband, mother, and sister (red arrow), but not in the father, consistent with autosomal dominant inheritance. This variant has not been previously reported in HS or other diseases, suggesting it may be pathogenic for HS in this family.

**Table 2:** Summary of clinical phenotypes observed in the proband and family members.

Relationship to the proband	Gender	Age	Clinical manifestations
Proband	Female	9	Mild microcytic anemia, clinically apparent jaundice, mild splenomegaly
Mother	Female	36	Mild microcytic anemia before splenectomy, normal hemoglobin level after splenectomy, no jaundice
Sister	Female	14	Mild microcytic anemia, subclinical jaundice, mild splenomegaly
Father	Male	47	Asymptomatic

### 3.2. Peripheral Blood Smear and SEM Analysis of *Ank1* c.5198G>A Knock‐In Mice

To investigate the pathogenicity of *ANK1* c.5096G>A (p.R1699K), an *Ank1* c.5198G>A (p.R1733K) knock‐in mouse model was generated (Figure 1a–d). Wright–Giemsa staining of peripheral blood smears showed WT RBCs had a typical biconcave disc shape, whereas mutant RBCs had significantly increased spherocytes (round, nonbiconcave, no central pallor, a key HS hallmark) (Figure 1e,f). SEM confirmed this: WT RBCs maintained biconcavity, whereas mutant RBCs lost central pallor and adopted a rounded, spherocyte‐like shape (Figure 1g,h).

### 3.3. Hematological Analysis of *Ank1* c.5198G>A Knock‐In Mice

CBC analysis (Table 3) showed mutant mice had significantly lower mean corpuscular volume (MCV), mean corpuscular hemoglobin (MCH), and mean corpuscular hemoglobin concentration (MCHC) than WT mice (*p* < 0.05, *t*‐test), indicating microcytosis with HS‐like membrane abnormalities without overt anemia (hemoglobin and hematocrit were not significantly decreased). RBC and hematocrit (HCT) were slightly elevated (not statistically significant); PLT and WBC counts were normal. Welch′s *t*‐test confirmed reduced MCV, MCH, and MCHC in mutants, verifying RBC membrane instability. Reticulocyte count was significantly higher in mutant mice (19.34 ± 13.42 × 10^10^/L) compared with WT (15.23 ± 5.16 × 10^10^/L, *p* = 0.049), whereas reticulocyte percentage showed no significant difference (1.84*%* ± 1.47*%* vs 1.52*%* ± 0.57*%*, *p* = 0.122).

**Table 3:** Hematological parameters and spleen morphology in wild‐type and *Ank1* c.5198G>A knock‐in mice (*m* *e* *a* *n* ± *S* *D*).

Parameter	Wild‐type (*n* = 24)	Mutant (*n* = 49)	*p*
RBC (×10^6^/*μ*L)	10.41 ± 0.88	10.74 ± 1.02	0.065
HGB (g/L)	157.8 ± 9.5	159.7 ± 11.2	0.091
HCT (%)	52.1 ± 3.4	53.5 ± 3.9	0.078
MCV (fL)	50.9 ± 1.5	48.3 ± 1.8	*0.002*
MCH (pg)	15.2 ± 0.4	14.6 ± 0.5	*0.015*
MCHC (g/L)	302 ± 8	296 ± 10	*0.038*
RDW (%)	15.8 ± 1.2	15.5 ± 1.4	0.112
WBC (×10^3^/*μ*L)	12.3 ± 3.2	13.1 ± 4.0	0.289
PLT (×10^3^/*μ*L)	1032 ± 110	1067 ± 122	0.351
Ret (%)	1.52 ± 0.57	1.84 ± 1.47	0.122
Ret (×10^10^/L)	15.23 ± 5.16	19.34 ± 13.42	*0.049*
Splenic sinus (%)	29.17 ± 4.23	36.08 ± 4.70	*0.032*
Splenic cord (%)	46.28 ± 2.11	43.20 ± 2.01	*0.035*
Splenic sinus/splenic cord	0.63 ± 0.12	0.84 ± 0.14	*0.026*
Splenic white pulp (%)	24.52 ± 2.94	20.72 + 3.6	*0.091*

*Note:* Italicized values denote   *p* < 0.05.

Abbreviations: HCT, hematocrit; HGB, hemoglobin; HS, hereditary spherocytosis; MCH, mean corpuscular hemoglobin; MCHC, mean corpuscular hemoglobin concentration; MCV, mean corpuscular volume; PLT, platelet; RBC, red blood cell; Ret, reticulocyte; RDW, red cell distribution width; WBC, white blood cell.

### 3.4. Osmotic Fragility Test of *Ank1* c.5198G>A Knock‐In Mice

**Figure 3:**
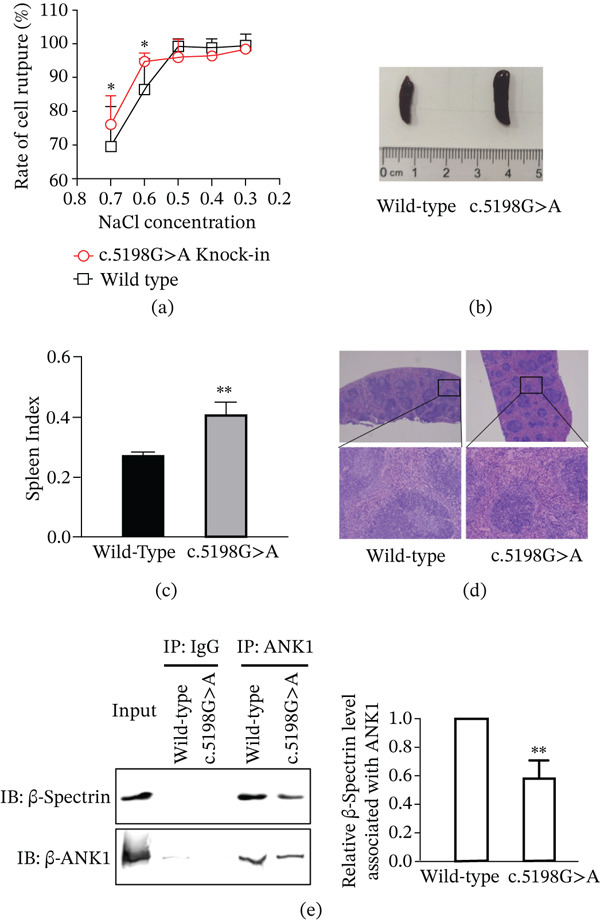
Functional and mechanistic characterization of *Ank1* c.5198G>A (p.R1733K) knock‐in mice. (a) Osmotic fragility assay.  ^∗^ *p* < 0.05 compared with the wild‐type group (*n* = 6 per group, two‐way ANOVA with Sidak′s posttest). (b) Representative gross images of spleens from wild‐type and *Ank1* c.5198G>A knock‐in mice. (c) Quantitative spleen index (spleen weight/body weight × 100%) in knock‐in (*n* = 23) and wild‐type (*n* = 23) mice.  ^∗∗^ *p* < 0.01, unpaired *t*‐test. (d) Representative H&E–stained spleen sections from wild‐type and *Ank1* c.5198G>A knock‐in mice. (e) Coimmunoprecipitation (co‐IP) assay for ankyrin‐1 and *β*‐spectrin. Left panel: Immunoblot of input lysates (total protein). Right panel: Immunoblot of anti–ankyrin‐1 immunoprecipitates (IP: And1). Bar graph shows densitometric quantification of *β*‐spectrin band intensity normalized to input (*n* = 3 independent experiments). Data are mean ± SD.  ^∗∗^ *p* < 0.01, unpaired *t*‐test. IP, immunoprecipitated; IB, immunoblot.

Osmotic fragility assays (assessing RBC membrane stability under hypotonic stress) showed mutant RBCs had significantly enhanced hemolysis at 0.7% and 0.6% NaCl concentrations compared with WT RBCs (*p* < 0.05, Figure 3a), consistent with HS characteristics. This is attributed to the reduced surface area‐to‐volume ratio and increased rigidity of spherocytes, which impair their resistance to osmotic pressure changes.

### 3.5. Spleen Morphology and Spleen Index of Ank1 R1733K Mutant Mice

Gross and histological analysis of spleens showed visible splenomegaly in mutant mice (Figure 3b). Quantitative assessment of the spleen index (spleen weight/body weight × 100*%*) revealed mutant mice (*n* = 23, 0.3577 ± 0.0888) had a significantly higher index than WT mice (*n* = 23, 0.2784 ± 0.0569) (*p* = 0.0008, Figure 3c). H&E staining of spleen sections showed marked expansion of the red pulp, increased extramedullary hematopoiesis, and abundant hemosiderin‐laden macrophages with erythrophagocytosis in mutant mice, consistent with compensatory response to hemolysis (Figure 3d and Table 3). This suggests the Ank1 R1733K mutation promotes increased splenic sequestration of RBCs or erythrophagocytosis, a potential compensatory response to excess spherocytes.

### 3.6. EMA Flow Cytometry of *Ank1* c.5198G>A (p.R1733K) Knock‐In Mice

Flow cytometry was used to measure EMA fluorescence intensity (EMA specifically binds band 3, a key RBC membrane protein; reduced intensity indicates band 3 deficiency, an HS hallmark). Mutant mice (*n* = 7) had a significant reduction in EMA fluorescence intensity (−20.36% to −27.60% vs. normal controls), whereas WT mice (*n* = 4) showed an increase (+8.00% to +12.00%) (Table 4). A Mann–Whitney *U* test confirmed statistical significance (*p* < 0.01), indicating disrupted band 3 expression and impaired RBC membrane stability.

**Table 4:** EMA fluorescence intensity between wild‐type and *Ank1* c.5198G>A knock‐in mice (*m* *e* *a* *n* ± *S* *D*).

	EMA fluorescence intensity (%)	*p*
Mutant (*n* = 7)	−23.55 ± 7.84	*p* < 0.01
Wild type (*n* = 4)	+10.00 ± 2.00	

Abbreviation: EMA, eosin‐5 ^′^‐maleimide.

### 3.7. Co‐IP of *Ank1* c.5198G>A (p.R1733K) Mutant Mice

Co‐IP with antiankyrin antibodies was performed to assess the ankyrin/*β*‐spectrin interaction. In WT mice, *β*‐spectrin was efficiently coimmunoprecipitated with ankyrin (confirming a stable complex); in heterozygous Ank1 R1733K mutant mice, significantly less *β*‐spectrin was pulled down (Figure 3e). Densitometric analysis of three independent co‐IP experiments revealed that the amount of *β*‐spectrin coprecipitated with ankyrin‐1 was reduced by 41*%* ± 11.8*%* in mutant mice compared with WT mice (*p* < 0.01, unpaired *t*‐test). This indicates the mutation weakens ankyrin–*β*‐spectrin binding, which may contribute to membrane instability and the spherocytic phenotype in mutant RBCs.

## 4. Discussion

This study analyzed Ank1 R1733K mutant mice, identifying core HS‐related phenotypes: reduced MCV, MCH, and MCHC (consistent with microcytosis without overt anemia); increased RBC hemolysis at 0.7%/0.6% NaCl (*p* < 0.05, confirming membrane fragility); spherocytes (loss of central pallor, spherical shape) via smears and SEM; reduced EMA fluorescence (*p* < 0.01, reflecting band 3 instability); and significant splenomegaly. Co‐IP further showed weakened ankyrin–*β*‐spectrin interaction in heterozygotes, clarifying membrane instability mechanisms.

HS is an inherited hemolytic anemia caused by erythrocyte membrane protein defects. The *ANK1* gene‐encoding ankyrin‐1 (which stabilizes the erythrocyte cytoskeleton by linking spectrin and band 3) is the most commonly mutated in HS [2, 5]. This study identifies a novel Ank1 R1733K mutation and provides functional evidence supporting its pathogenicity via a CRISPR/Cas9 knock‐in mouse model. Mutant mice′s reduced MCV/MCH/MCHC aligns with progressive RBC membrane loss in human HS [6–8]. Preserved hemoglobin/hematocrit suggests mild‐to‐moderate HS (distinct from severe Ank1‐null phenotypes), likely due to retained partial ankyrin‐1 function (evidenced by moderate EMA reduction). Increased osmotic fragility confirms membrane instability, consistent with prior findings that Ank1 mutations weaken cytoskeletal anchoring [9, 10]. The spherocyte phenotype (validated by microscopy) directly indicates disruption of the spectrin–ankyrin–band 3 complex‐key for erythrocyte shape‐driving vesiculation, membrane loss, and premature clearance (HS hallmarks). Reduced EMA fluorescence (band 3 instability) aligns with reports that Ank1 mutations disrupt spectrin–ankyrin–band 3 interactions, causing secondary band 3 defects [11], underscoring ankyrin‐1′s role in membrane protein integrity. Splenomegaly in mutants matches classic HS pathophysiology (splenic macrophages clear spherocytes [7, 12, 13]), and the H&E histology further supports active erythrophagocytosis and compensatory extramedullary hematopoiesis. A critical mechanistic finding: impaired ankyrin‐*β*‐Spectrin interaction. Ankyrin links the spectrin‐actin cytoskeleton to membrane proteins (essential for RBC shape/resilience [2, 5]); even subtle binding disruptions reduce deformability, trigger microvesiculation, and accelerate clearance [14]. The R1733K substitution lies near ankyrin′s C‐terminal regulatory domain (modulates spectrin binding [10, 15]), likely weakening this interaction and causing cytoskeletal uncoupling/spherocytes. The densitometric quantification showing a 41% reduction in binding provides strong support for this mechanism.

In conclusion, this study provides the first functional evidence supporting Ank1 R1733K pathogenicity, demonstrating its role in RBC membrane instability, microcytosis, and increased osmotic fragility. The knock‐in mouse model recapitulates key HS phenotypes, serving as a valuable preclinical tool. Our findings advance understanding of ANK1‐associated HS pathogenesis and provide a foundation for future mechanistic studies. Limitations: small sample sizes for some analyses (e.g., histology, flow cytometry, limiting generalizability), incomplete characterization of downstream signaling/compensatory mechanisms, and need for biophysical/proteomic studies to define R1733K′s precise molecular effects.

## Author Contributions

Chen Guang, Ding Xiaofei, and Chen Jie conceived and designed the study. Xu Linglong and Gao Jingwen developed the methodology. Gao Jingwen, Ding Xiaofei, Zhu Wenting, Lu Nina, Zhao Lei, Guo Yanrong, Ding Xiaoxiao, Zhou Siyun, Cai Zihao, Zhong Zhengyang, Zhu Bo, Chen Sai, Chen Jianlin, and Chen Jie acquired the data (i.e., provided animals, acquired and managed patients, and provided facilities). Xu Linglong, Ding Xiaofei, Chen Jie, and Chen Guang analyzed and interpreted the data (e.g., statistical analysis). Xu Linglong and Chen Guang confirm the authenticity of all the raw data. Xu Linglong and Gao Jingwen contributed equally to this work.

## Funding

This study was supported by National Natural Science Foundation of China (82473959); Zhejiang Provincial Natural Science Foundation (LBY23H200004, LHDMY22H310001); and Taizhou Technology Project (24ywa14).

## Disclosure

All authors have read and approved the final manuscript.

## Ethics Statement

All animal procedures complied with regulations of the Taizhou University Medical School Animal Center and the Institutional Animal Care and Use Committee (TZXY‐2023‐20231095).

## Consent

Participants gave informed consent for this study, which was approved by the Institutional Review Board of Taizhou Central Hospital (Approval No. 2024‐07‐39; Taizhou, China).

## Conflicts of Interest

The authors declare no conflicts of interest.

## Supporting information


**Supporting Information** Additional supporting information can be found online in the Supporting Information section.  

## Data Availability

Data are available on request due to privacy/ethical restrictions.
