# Supplementary material for: A Pathogenic ANK1 c.5096G>A Mutation Disrupting the Ankyrin‐1/β‐Spectrin Interaction in Hereditary Spherocytosis
Source: Hum Mutat. 2026 Aug 19;2026:7320235. doi: 10.1155/humu/7320235 (PMC13487465; doi:10.1155/humu/7320235)
Supplement: Supplementary file 1 [file HUMU-2026-7320235-s001.pdf]

European Journal of Haematology

Original Article

Title：A novel c.5096G>A mutation in the Ankyrin-1(Ank1) gene Result in hereditary spherocytosis (HS) by disrupting this interaction between Ank1 and β-Spectrin (SPTB)

Submission ID 05bfb65c-3904-43a7-a633-450320f9d460

Submission Version Initial Submission

PDF Generation 07 Oct 2025 07:49:41 EST by Atypon ReX

Authors

Dr. Xu Linglong

Affiliations

- Department of Hematology, Taizhou Central Hospital (Taizhou University Hospital), School of Medicine, Taizhou University, Taizhou, Zhejiang 318000, P. R. China

Dr. Gao Jingwen

Affiliations

- Department of Pharmacology, School of Medicine, Taizhou University, Taizhou, Zhejiang 318000, P. R. China

Dr. Zhu Wenting

ORCiD
https://orcid.org/0009-0003-5251-4644

Affiliations

- Department of Hematology, Taizhou Central Hospital (Taizhou University Hospital), School of Medicine, Taizhou University, Taizhou, Zhejiang 318000, P. R. China

Dr. Lu Nina

Affiliations

- Department of Hematology, Taizhou Central Hospital (Taizhou University Hospital), School of Medicine, Taizhou University, Taizhou, Zhejiang 318000, P. R. China

Dr. Ding Xiaoxiao

Affiliations

- Department of Hematology, Taizhou Central Hospital (Taizhou University Hospital), School of Medicine, Taizhou University, Taizhou, Zhejiang 318000, P. R. China

Dr. Zhao Lei

Affiliations

- Department of Laboratory Medicine, Taizhou Central Hospital (Taizhou University Hospital), School of Medicine, Taizhou University, Taizhou, Zhejiang 318000, P. R. China

Miss Zhou Siyun

Affiliations

- Zhejiang Key Laboratory of New Drug Development for Central Nervous System Diseases, Taizhou University, Zhejiang 318000, P. R. China
- Department of Pharmacology, School of Medicine, Taizhou University, Taizhou, Zhejiang 318000, P. R. China

Mr. Cai Zihao

Affiliations

- Zhejiang Key Laboratory of New Drug Development for Central Nervous System Diseases, Taizhou University, Zhejiang 318000, P. R. China
- Department of Pharmacology, School of Medicine, Taizhou University, Taizhou, Zhejiang 318000, P. R. China

Mr. Zhong Zhengyang

ORCiD
https://orcid.org/0000-0002-8555-6109

Affiliations

- Zhejiang Key Laboratory of New Drug Development for Central Nervous System Diseases, Taizhou University, Zhejiang 318000, P. R. China
- Department of Pharmacology, School of Medicine, Taizhou University, Taizhou, Zhejiang 318000, P. R. China

Mr. Zhu Bo

Affiliations

- Zhejiang Key Laboratory of New Drug Development for Central Nervous System Diseases, Taizhou University, Zhejiang 318000, P. R. China
- Department of Pharmacology, School of Medicine, Taizhou University, Taizhou, Zhejiang 318000, P. R. China

Dr. Chen Sai

Affiliations

- Department of Hematology, Taizhou Central Hospital (Taizhou University Hospital), School of Medicine, Taizhou University, Taizhou, Zhejiang 318000, P. R. China

Dr. Guo Yanrong

Affiliations

- Department of Hematology, Taizhou Central Hospital (Taizhou University Hospital), School of Medicine, Taizhou University, Taizhou, Zhejiang 318000, P. R. China

Dr. Chen Jianlin

Affiliations

- Department of Hematology, Taizhou Central Hospital (Taizhou University Hospital), School of Medicine, Taizhou University, Taizhou, Zhejiang 318000, P. R. China

Mrs. Chen Jie

Affiliations

- Department of Pharmacology, School of Medicine, Taizhou University, Taizhou, Zhejiang 318000, P. R. China

Prof. Ding Xiaofei

Affiliations

- Zhejiang Key Laboratory of New Drug Development for Central Nervous System Diseases, Taizhou University, Zhejiang 318000, P. R. China

Prof. Chen Guang
Corresponding Author
Submitting Author

ORCiD
https://orcid.org/0000-0002-1885-7374

Affiliations

- Department of Hematology, Taizhou Central Hospital (Taizhou University Hospital), School of Medicine, Taizhou University, Taizhou, Zhejiang 318000, P. R. China
- Zhejiang Key Laboratory of New Drug Development for Central Nervous System Diseases, Taizhou University, Zhejiang 318000, P. R. China
- Department of Pharmacology, School of Medicine, Taizhou University, Taizhou, Zhejiang 318000, P. R. China

Files for peer review

All files submitted by the author for peer review are listed below. Files that could not be converted to PDF are indicated; reviewers are able to access them online.

Name	Type of File	Size	Page
Manuscript-EJH.docx	Main Document - MS Word	2.0 MB	Page 5

Title： A novel c.5096G>A mutation in the Ankyrin-1(Ank1) gene Result in hereditary spherocytosis (HS) by disrupting this interaction between Ank1 and β-Spectrin (SPTB)

Xu Linglong1△, Gao Jingwen2, 3 △, Zhu Wenting1, Lu Nina1, Ding Xiaoxiao1, Zhao Lei4, Zhou Siyun1,2, Cai Zihao1,2, Zhong Zhengyang1,2, Zhu Bo1,2, Chen Sai1, Guo Yanrong1, Chen Jianlin1, Chen Jie 2, 3*, Ding Xiaofei2*, Chen Guang1,2, 3 *

1 Department of Hematology, Taizhou Central Hospital (Taizhou University Hospital), School of Medicine, Taizhou University, Taizhou, Zhejiang 318000, P. R. China.

2 Zhejiang Key Laboratory of New Drug Development for Central Nervous System Diseases, Taizhou University, Zhejiang 318000, P. R. China.

3 Department of Pharmacology, School of Medicine, Taizhou University, Taizhou, Zhejiang 318000, P. R. China.

4 Department of Laboratory Medicine, Taizhou Central Hospital (Taizhou University Hospital), School of Medicine, Taizhou University, Taizhou, Zhejiang 318000, P. R. China.

△These authors contributed equally to this work.

*Correspondence to Corresponding author: Chen Jie, Ding Xiaofei and Chen Guang, 1139 Shi-Fu Avenue, Department of pharmacology, School of Medicine, Taizhou University, Taizhou, Zhejiang, 318000, China. email: jchen@tzc.edu.cn, dxfei@tzc.edu.cn and gchen@tzc.edu.cn. Tel/Fax: 0576-88665198.

Abstract word count: 199

Manuscript word Count: 2334

Number of references: 16

Number of figures: 3

Number of tables: 4

Number of supplemental illustrations/tables: No

Keywords: hereditary spherocytosis (HS), Ankyrin-1 (Ank1), Ank1 c.5096G>A mutation, β-Spectrin

Conflicts of Interest: The authors have no conflicts of interest to disclose.

Novelty Statement

1. We identified a novel Ank1 mutation (c.5096G>A, p.R1699K) in a patient with classic HS phenotypes and a family history of hemolytic anemia
2. Our work validates the pathogenicity of the Ank1 c.5096G>A mutation in animal model and the patient.
3. Our work offers a valuable platform for further investigating the molecular mechanisms and diagnostic markers of HS.

Abstract: Hereditary spherocytosis (HS) is a common inherited hemolytic anemia characterized by spherical erythrocytes, splenomegaly, and increased osmotic fragility, with Ank1 mutations being the most frequent cause. We identified a novel Ank1 mutation (c.5096G>A, p.R1699K) in a patient with classic HS phenotypes and a family history of hemolytic anemia. To explore its pathogenicity, we generated a CRISPR/Cas9-mediated knock-in mouse carrying the homologous Ank1 c.5198G>A (R1733K) on a C57BL/6J background, followed by a series of hematological and morphological analyses. Compared with wild-type mice, the knock-in mice displayed typical HS features. Hematologically, they showed reduced MCV and elevated MCHC. The EMA fluorescence intensity was significantly decreased, and RBCs exhibited increased osmotic fragility. Morphologically, peripheral smears revealed abundant spherocytes, and electron microscopy showed a transformation from normal biconcave discs to spherical erythrocytes. Additionally, the knock-in mice presented with significant splenomegaly. Mechanistically, co-immunoprecipitation analysis indicated a weakened association between Ankyrin-1 and Spectrin in the knock-in mice. This study provides in vivo evidence that Ank1 R1733K mutation impairs erythrocyte membrane stability and cytoskeletal integrity, recapitulating the HS phenotype in mice. It validates the pathogenicity of the Ank1 c.5096G>A mutation in the patient and offers a valuable platform for further investigating the molecular mechanisms of HS.

1. Introduction

Hereditary spherocytosis (HS) is a common inherited hemolytic anemia, characterized by spherocytes—osmotically fragile red blood cells (RBCs) prone to premature splenic destruction[1]. It arises from mutations in genes encoding key erythrocyte membrane proteins (Ank1, SPTB, SPTA1, EPB42, SLC4A1) that maintain RBC membrane integrity[2, 3]. Among these, Ank1 mutations are most prevalent (40-65% of HS cases); they disrupt the Ankyrin-Spectrin-band 3 network, causing membrane instability, increased fragility, and hemolysis[4, 5].

The Ank1 gene (chromosome 8p11.21) contains 42 exons and encodes the 210-kDa Ankyrin-R, a central scaffold of the erythrocyte membrane skeleton[4]. Its function relies on three domains: an N-terminal membrane-binding domain (24 Ankyrin repeats, binds band 3/RhAG), a central Spectrin-binding domain (ZU5-UPA-ZU5 modules, anchors β-Spectrin dimers), and a C-terminal regulatory domain (governs protein stability). During erythropoiesis, Ank1 mediates vertical integration of the membrane skeleton: its N-terminus tethers band 3 tetramers, while the central domain links the β-Spectrin–actin horizontal network, forming a "sandwich" structure that ensures RBC biconcavity and deformability. Physiologically, Ank1 maintains membrane integrity via lipid bilayer-cytoskeleton coupling (preventing vesiculation) and stabilizes surface antigens; it also modulates RBC metabolism by interacting with pyruvate kinase (PKLR) to regulate glycolysis for ATP-dependent membrane stabilization[5]. Ank1 deficiency disrupts vertical linkages, causing progressive membrane loss and morphological abnormalities.

Ank1 mutations cause 40-50% of HS cases, with >200 pathogenic variants reported. Major types include nonsense (30%), frameshift (25%), splice-site (20%), missense (15%), and large deletions (10%), with hotspots in exon 8 (Ankyrin repeats), exons 13-20 (Spectrin-binding domain), and exon 37

(regulatory domain). Nonsense/frameshift mutations (e.g., c.648G>A/p.Trp216*, c.3614_3617delCTTG/p.Ser1205Tyrfs*31) produce truncated proteins with dominant-negative effects; splice-site mutations (e.g., intron 29 c.4570-1G>A) cause exon skipping; missense mutations (e.g., exon 13 c.1424T>C/p.Leu475Pro) reduce Spectrin affinity; promoter deletions decrease Ank1 expression by >50%. These defects uncouple the cytoskeleton from the lipid bilayer, causing membrane microvesiculation, surface area loss, and rigid spherocytes—these cells undergo splenic sequestration, leading to chronic extravascular hemolysis[4].

In this study, we identified an Ank1 c.5096G>A mutation in a clinical HS patient and investigated its pathogenic role. Using CRISPR/Cas9, we introduced the homologous Ank1 c.5198G>A mutation into C57BL/6J mice to establish a knock-in model. We aimed to determine if this mutation alone induces spherocytosis, membrane instability, and hemolytic anemia—providing the first functional validation of Ank1 c.5198G>A in HS pathogenesis. Findings will enhance understanding of HS molecular mechanisms and inform refined genetic diagnosis and targeted therapies.

2. Materials and Methods

2.1 Participants

Participants gave informed consent for this study, which was approved by the Institutional Review Board of Taizhou Central Hospital (approval no. 2024-07-39; Taizhou, China).

2.2 Genetic analysis

Peripheral blood (3 mL) was collected from a female HS patient and her family. Genomic DNA (gDNA) was extracted from peripheral blood leukocytes using a MagBead-based kit (Tiangen Biotech, Beijing); its concentration was measured with a Qubit 3.0 fluorometer (Invitrogen, USA) and integrity verified via 1% agarose gel electrophoresis.

Qualified gDNA was sheared using the VAHTS® Universal DNA Library Kit (Vazyme, Nanjing), followed by liquid-phase capture with the xGen® Exome Research Panel v2.0 (Integrated DNA Technologies, USA) to construct a whole-exome library. The library was sequenced on the MGISEQ-T7 platform (BGI Genomics) for 150 bp paired-end whole-exome sequencing (experiments conducted by Wuhan Primbio Medical Laboratory).

Bioinformatics analysis showed the patient's sample generated >10 Gb data, with >99% of regions having >20× coverage and average depth >12×. Reads were aligned to the GRCh37/hg19 genome using BWA (v0.7.16a); low-quality reads/adapters and PCR duplicates were removed with fastp (v0.21.0) and sambamba (v0.8.0), respectively. Variant detection (SNVs, small Indels <50 bp) was performed on BAM files via GATK (v4.0.8.1). Variants were filtered by depth (>20×) and quality (QualByDepth>2), then annotated with ANNOVAR (v20200608). Further filtering prioritized minor allele frequency, protein effects, in silico scores, disease genetic patterns, and clinical phenotype consistency. Recessive splicing sites were predicted using HSF (v3.1), VarSEAK, and SpliceAI. Candidate variant pathogenicity was interpreted per ACMG/AMP and ClinGen SVI guidelines, and validated by Sanger sequencing.

2.3 Generation of Ank1 c.5198G>A Knock-In Mice

C57BL/6J mice were obtained from the Shanghai Model Organisms Center. All animal procedures complied with regulations of the Taizhou University Medical School Animal Center and the Institutional Animal Care and Use Committee (TZXY-2023-20231095). Mice were housed in a specific pathogen-free facility under a 12-h light/12-h dark cycle, with free access to food and water.

Heterozygous Ank1 c.5198G>A (p.R1733K) knock-in mice (targeting murine Ank1, ENSMUSG00000031543) were generated by Shanghai Model Organisms (Shanghai, China). The

targeting guide RNA sequence was 5’-ACCCACCGAGCATCCTTACC-3’. In vitro transcribed Cas9 mRNA, chimeric sgRNA, and a 241-bp single-stranded oligodeoxynucleotide (ssODN) were injected into C57BL/6 mouse zygotes (Fig. 2A). F0 mice were validated by sequencing using primers: mAnk1-F (5’-CAGATGCCACAAATGGCCTG-3’) and mAnk1-R (5’-ACGGTACCATGTCCCAAACC-3’). Mice with the expected single-nucleotide mutation were crossed with wild-type (WT, +/+) C57BL/6 mice to generate F1 knock-in mice. The ssODN sequence was: 5’-TCTCAGGCACAGAGCAGGACACGGAGACTGAAGTGTCTCTTGTTTCAGGCCAGCAGCGAGTTCACGCCCGAATCACAGACTCACCCTCAGTGAGGCAGGTGCTGGACAGAAGCCAGGCCAAGTAAGGATGCTCGGTGGGTCTCTTCTGTCTTCCTGGACTTTGGGGCCAGCTATCCTGGCACTGGGCGCAGGCAGAAGGTTTGGATGCTGCTCTTCATCCTGCATTGTAAACACGAATGCC-3’.

2.4 Hematological Analysis

To assess hematological parameters, whole blood was collected via retro-orbital bleeding and analyzed using an automated hematology analyzer by following the instrument manual.

2.5 EMA Flow Cytometry

Freshly isolated RBCs (5 μL) were incubated in 200 μL of EMA dye solution for 1 hour in the dark at room temperature. After incubation, cells were washed three times with PBS and resuspended in 500 μL PBS for flow cytometric analysis. Fluorescence intensity (mean fluorescence intensity, MFI) was measured using a BD FACSCalibur flow cytometer.

2.4 Osmotic Fragility Assay

RBCs from wild-type and Ank1 c.5198G>A mutation (R1733K) mice were incubated in NaCl solutions ranging from 0.7% to 0.3% for 30 minutes at room temperature. After centrifugation, the

supernatant absorbance was measured at 540 nm. Hemolysis (%) was calculated relative to complete lysis (100%) in distilled water.

2.5 Peripheral Blood Smears and Scanning Electron Microscopy (SEM)

Thin blood smears were prepared from the EDTA-treated blood samples and the Wright–Giemsa staining method with methanol, thiazine and eosin dyes (Baso Diagnostics Inc., Zhuhai, China). Olympus BX35 microscope (Tokyo, Japan) with LABB C1C22 lens was used to take photographs (Beion v 4.20). Smears were examined under light microscopy at 1000× magnification**.**

For SEM Imaging of RBC Morphology, freshly isolated RBCs were fixed with 2.5% glutaraldehyde, dehydrated in ethanol, and coated with gold-palladium. Imaging was performed using a scanning electron microscope at 5 kV acceleration voltage.

2.8 Co-immunoprecipitation (co-IP) assay

Red blood cells were lysed in ice-cold RIPA buffer (Beyotime Biotechnology, Haimen) with protease/phosphatase inhibitors (Yeasen, Shanghai), after which lysates were clarified by centrifugation (12,000g, 15 min, 4°C). Protein concentration was measured via BCA kit (Pierce), and for each sample, 500 μg total protein was incubated overnight at 4°C with 2 μg anti-Ankyrin-1 antibody (Santa Cruz, sc-376187) or isotype IgG, then with 30 μL protein A/G magnetic beads (Beyotime, Haimen) overnight at 4°C with gentle rotation. The beads were washed three times with lysis buffer to remove non-specific proteins, and bound proteins were eluted by boiling in SDS loading buffer, run on SDS-PAGE, and immunoblotted with anti-Spectrin antibody (Abcam, ab129065). Finally, blots were developed with ECL reagents (Biosharp, BL523B), and images captured using GelView 6000 Plus (Guangzhou Biolight).

2.9 Statistical Analysis

All data were expressed as mean ± standard deviation (SD). Statistical significance was determined using, Welch's t-test for normally distributed data, Mann-Whitney U test for non-normally distributed data. $p < 0.05$ was considered statistically significant. All analyses were performed using GraphPad Prism 10.

3. Results

3.1 Clinical and genetic findings of the proband and her family members

A 9-year-old female proband (born 2014) was admitted for persistent jaundice, darkened urine, anorexia, and nausea. Physical examination showed mild jaundice and splenomegaly (spleen palpable 1 cm below costal margin); no other abnormalities were found.

Complete blood count (CBC) results: white blood cell count 5.9×10^{9}/L, red blood cell (RBC) count 4.09×10^{12}/L, hemoglobin 118 g/L, platelet count 389×10^{9}/L. Liver function tests revealed elevated total bilirubin (115.5 μmol/L), with direct bilirubin 45.3 μmol/L and indirect bilirubin 70.2 μmol/L. Hemolysis-related tests showed increased RBC osmotic fragility and reduced mean fluorescence intensity (MFI) in the erythrocyte eosin-5-maleimide (EMA) binding assay (Table 1).

The proband had a positive family history of inherited hematologic disorders: her maternal grandmother was diagnosed with hereditary spherocytosis (HS), her mother had splenomegaly and a splenectomy history, and her elder sister also had splenomegaly (Table 2). Combined with clinical and laboratory findings, HS was suspected. Peripheral blood smear and whole-exome sequencing (WES) were performed for the proband and her family (Fig. 1).

Peripheral blood smears showed normal RBC morphology in the father, while spherocytes were observed in the mother, proband, and elder sister. Sanger sequencing identified a novel Ank1 variant (c.5096G>A, p.R1699K) in the proband, mother, and sister (red arrow), but not in the father—consistent

with autosomal dominant inheritance. This variant has not been previously reported in HS or other diseases, suggesting it may be pathogenic for HS in this family.

3.2 Peripheral Blood Smear and SEM Analysis of Ank1 c.5198G>A Knock-In Mice

To verify the pathogenicity of Ank1 c.5096G>A (p.R1699K), an Ank1 c.5198G>A (p.R1733K) knock-in mouse model was generated (Fig. 2a-2d). Wright-Giemsa staining of peripheral blood smears showed wild-type RBCs had a typical biconcave disc shape, while mutant RBCs had significantly increased spherocytes (round, non-biconcave, no central pallor— a key HS hallmark) (Fig. 2e, 2f). Scanning electron microscopy (SEM) confirmed this: wild-type RBCs maintained biconcavity, while mutant RBCs lost central pallor and adopted a rounded, spherocyte-like shape (Fig. 2g, 2h).

3.3 Hematological Analysis of Ank1 c.5198G>A Knock-in Mice

CBC analysis (Table 3) showed mutant mice had significantly lower mean corpuscular volume (MCV), mean corpuscular hemoglobin (MCH), and mean corpuscular hemoglobin concentration (MCHC) than wild-type mice ($p < 0.05$, t-test), indicating microcytic anemia. Red blood cell count (RBC) and hematocrit (HCT) were slightly elevated (not statistically significant); platelet (PLT) and white blood cell (WBC) counts were normal. Welch's t-test confirmed reduced MCV, MCH, and MCHC in mutants, verifying RBC membrane instability.

3.4 Osmotic Fragility Test of Ank1 c.5198G>A Knock-in Mice

Osmotic fragility assays (assessing RBC membrane stability under hypotonic stress) showed mutant RBCs had significantly enhanced hemolysis at 0.7% and 0.6% NaCl concentrations compared to wild-type RBCs ($p < 0.05$, Fig. 3A)—consistent with HS characteristics. This is attributed to the reduced surface area-to-volume ratio and increased rigidity of spherocytes, which impair their resistance to osmotic pressure changes.

3.5 Spleen Morphology and Spleen Index of Ank1 R1733K Mutant Mice

Gross and histological analysis of spleens showed visible splenomegaly in mutant mice (Fig. 3b). Quantitative assessment of the spleen index (spleen weight/body weight × 100%) revealed mutant mice (n=23, 0.3577 ± 0.08880) had a significantly higher index than wild-type mice (n=23, 0.2784±0.05695) (p=0.0008, Fig. 3c). This suggests the Ank1 R1733K mutation promotes increased splenic sequestration of RBCs or erythrophagocytosis, a potential compensatory response to excess spherocytes.

3.6 EMA Flow Cytometry of Ank1 c.5198G>A (p.R1733K) Knock-in Mice

Flow cytometry was used to measure EMA fluorescence intensity (EMA specifically binds band 3, a key RBC membrane protein; reduced intensity indicates band 3 deficiency, an HS hallmark). Mutant mice (n=7) had a significant reduction in EMA fluorescence intensity (-20.36% to -27.60% vs. normal controls), while wild-type mice (n=4) showed an increase (+8.00% to +12.00%) (Table 4). A Mann-Whitney U test confirmed statistical significance ($p < 0.01$), indicating disrupted band 3 expression and impaired RBC membrane stability.

3.7 Co-immunoprecipitation of Ank1 c.5198G>A (p.R1733K) Mutant Mice

Co-immunoprecipitation (Co-IP) with anti-Ankyrin antibodies was performed to assess the Ankyrin/β-Spectrin interaction. In wild-type mice, β-Spectrin was efficiently co-immunoprecipitated with Ankyrin (confirming a stable complex); in heterozygous Ank1 R1733K mutant mice, significantly less β-Spectrin was pulled down (Fig. 3d). This indicates the mutation weakens Ankyrin-β-Spectrin binding, which may contribute to membrane instability and the spherocytic phenotype in mutant RBCs.

4. Discussion

This study analyzed Ank1 R1733K mutant mice, identifying core hereditary spherocytosis (HS)-related phenotypes: reduced mean corpuscular volume (MCV), mean corpuscular hemoglobin (MCH),

and mean corpuscular hemoglobin concentration (MCHC) (consistent with microcytic anemia, though hemoglobin/hematocrit remained normal, indicating mild-to-moderate HS); increased RBC hemolysis at 0.7%/0.6% NaCl ($p < 0.05$, confirming membrane fragility); spherocytes (loss of central pallor, spherical shape) via smears and scanning electron microscopy (SEM); reduced eosin-5-maleimide (EMA) fluorescence ($p < 0.01$, reflecting band 3 instability); and significant splenomegaly. Co-immunoprecipitation (Co-IP) further showed weakened Ankyrin-β-Spectrin interaction in heterozygotes, clarifying membrane instability mechanisms.

HS is an inherited hemolytic anemia caused by erythrocyte membrane protein defects. The Ank1 gene—encoding Ankyrin-1 (which stabilizes the erythrocyte cytoskeleton by linking Spectrin and band 3)—is the most commonly mutated in HS [2, 5]. This study identifies a novel Ank1 R1733K mutation and validates its function via a CRISPR/Cas9 knock-in mouse model. Mutant mice's reduced MCV/MCH/MCHC aligns with progressive RBC membrane loss in human HS [6-8]. Preserved hemoglobin/hematocrit suggests mild-to-moderate HS (distinct from severe Ank1-null phenotypes), likely due to retained partial Ankyrin-1 function (evidenced by moderate EMA reduction). Increased osmotic fragility confirms membrane instability, consistent with prior findings that Ank1 mutations weaken cytoskeletal anchoring [9, 10]. The spherocyte phenotype (validated by microscopy) directly indicates disruption of the Spectrin-Ankyrin-band 3 complex—key for erythrocyte shape—driving vesiculation, membrane loss, and premature clearance (HS hallmarks). Reduced EMA fluorescence (band 3 instability) aligns with reports that Ank1 mutations disrupt Spectrin-Ankyrin-band 3 interactions, causing secondary band 3 defects [12], underscoring Ankyrin-1's role in membrane protein integrity. Splenomegaly in mutants matches classic HS pathophysiology (splenic macrophages clear spherocytes [7, 13, 14]), validating the model for studying splenic involvement in hemolysis. A critical

mechanistic finding: impaired Ankyrin-β-Spectrin interaction. Ankyrin links the Spectrin-actin cytoskeleton to membrane proteins (essential for RBC shape/resilience [2, 5]); even subtle binding disruptions reduce deformability, trigger microvesiculation, and accelerate clearance [15]. The R1733K substitution lies near Ankyrin's C-terminal regulatory domain (modulates Spectrin binding [10, 16]), likely weakening this interaction and causing cytoskeletal uncoupling/spherocytes.

In conclusion, this study provides the first functional validation of Ank1 R1733K, demonstrating its role in RBC membrane instability, microcytosis, and increased osmotic fragility. The knock-in mouse model recapitulates key HS phenotypes, serving as a valuable preclinical tool. Findings advance understanding of Ank1-associated HS pathogenesis and inform future mechanistic/therapeutic studies. Limitations: small sample sizes for some analyses (e.g., histology, flow cytometry, limiting generalizability), incomplete characterization of downstream signaling/compensatory mechanisms, and need for biophysical/proteomic studies to define R1733K's precise molecular effects.

Data availability statement

Data is available upon request.

Funding statement

This research was supported by Natural Science Foundation of China under Grant No. 82473959. Zhejiang Provincial Natural Science Foundation of China under Grant No. LBY23H200004, LHDMY22H310001. Taizhou Technology Project 24ywa14.

Conflict of interest disclosure

Authers declare no conflict.

Ethics approval statement

All animal procedures complied with regulations of the Taizhou University Medical School Animal

Center and the Institutional Animal Care and Use Committee (TZXY-2023-20231095)

Patient consent statement

Participants gave informed consent for this study, which was approved by the Institutional Review Board of Taizhou Central Hospital (approval no. 2024-07-39; Taizhou, China).

Permission to reproduce material from other sources

N/A

Clinical trial registration

N/A

Author Contributions

CG, DXF and CJ conceived and designed the study. XLL and GJW developed the methodology, while GJW, DXF, ZWT, LNN, ZL, HYR, DXX, ZJF, ZXC, YLM and CJ acquired the data (i.e., provided animals, acquired and managed patients and provided facilities). XLL, DXF, CJ, ZJ and CG analyzed and interpreted the data (e.g., statistical analysis). XLL and CG confirm the authenticity of all the raw data. All authors have read and approved the final manuscript.

Artificial Intelligence Generated Content (AIGC) using statement

AI was used to check English grammar.

Reference

1. Bolton-Maggs, P.H., et al., *Guidelines for the diagnosis and management of hereditary spherocytosis.* Br J Haematol, 2004. **126**(4): p. 455-74.

2. Da Costa, L., et al., *Hereditary spherocytosis, elliptocytosis, and other red cell membrane disorders.* Blood Rev, 2013. **27**(4): p. 167-78.

3. Perrotta, S., P.G. Gallagher, and N. Mohandas, *Hereditary spherocytosis.* Lancet, 2008. **372**(9647): p. 1411-26.

4. Hauser, F., et al., *Hereditary Spherocytosis: Can Next-Generation Sequencing of the Five Most Frequently Affected Genes Replace Time-Consuming Functional Investigations?* Int J Mol Sci, 2023. **24**(23).

5. He, B.J., et al., *Molecular Genetic Mechanisms of Hereditary Spherocytosis: Current Perspectives.* Acta Haematol, 2018. **139**(1): p. 60-66.

6. Iolascon, A., R.A. Avvisati, and C. Piscopo, *Hereditary spherocytosis.* Transfus Clin Biol, 2010. **17**(3): p. 138-42.

7. Polizzi, A., et al., *Overview on Hereditary Spherocytosis Diagnosis.* Int J Lab Hematol, 2025. **47**(1): p. 18-25.

8. Tole, S., et al., *Genotype-phenotype correlation in children with hereditary spherocytosis.* Br J Haematol, 2020. **191**(3): p. 486-496.

9. Iolascon, A., I. Andolfo, and R. Russo, *Advances in understanding the pathogenesis of red cell membrane disorders.* Br J Haematol, 2019. **187**(1): p. 13-24.

10. Hao, L., et al., *Two novel Ank1 loss-of-function mutations in Chinese families with hereditary spherocytosis.* J Cell Mol Med, 2019. **23**(6): p. 4454-4463.

11. Kar, R., P. Mishra, and H.P. Pati, *Evaluation of eosin-5-maleimide flow cytometric test in diagnosis of hereditary spherocytosis.* Int J Lab Hematol, 2010. **32**(1 Pt 2): p. 8-16.

12. Van Zwieten, R., et al., *Hereditary spherocytosis due to band 3 deficiency: 15 novel mutations in SLC4A1.* Am J Hematol, 2013. **88**(2): p. 159-60.

13. Hughes, M.R., et al., *A novel ENU-generated truncation mutation lacking the Spectrin-binding*

and C-terminal regulatory domains of Ank1 models severe hemolytic hereditary spherocytosis. Exp Hematol, 2011. **39**(3): p. 305-20, 320 e1-2.

14. Kildey, K., et al., *Characterization of ENU-induced Mutations in Red Blood Cell Structural Proteins.* Comput Struct Biotechnol J, 2013. **6**: p. e201303012.

15. Vorselen, D., et al., *The fluid membrane determines mechanics of erythrocyte extracellular vesicles and is softened in hereditary spherocytosis.* Nat Commun, 2018. **9**(1): p. 4960.

16. Machnicka, B., et al., *Spherocytosis-Related L1340P Mutation in Ankyrin Affects Its Interactions with Spectrin.* Life (Basel), 2023. **13**(1).

Table1.Laboratory Findings of Hemolysis and Erythrocyte Membrane Stability in the Proband

Assay Name	Result	Reference Range
**1.Hemoglobin analysis**		
Anti-Alkaline Hemoglobin	1.10%	0-2.5%
Hemoglobin A_2	3.00%	2.5-3.5%
Isopropanol Test	negative	negative
Heinz Body Test	2%	0-1%
Hemoglobin H Inclusion Test	0%	0-5%
**2.Erythrocyte metabolic enzymes activity**		
Pyrimidine 5'-Nucleotidase	3.25	2.60-3.52
Glucose-6-Phosphate Dehydrogenase	1544 U/L	N/A
Pyruvate Kinase	normal	normal
Glucose Phosphate Isomerase	normal	normal
**3.Erythrocyte membrane stability assay**		
Acidified Glycerol Lysis	25 sec	>290 sec
Initial Hemolysis in Osmotic Fragility	0.60%	0.44-0.48%
Complete Hemolysis in Osmotic Fragility	0.40%	0.28-0.36%
Hyperosmotic Sucrose Lysis	8.40%	0-16.9%
Red Cell Incubation Osmotic Fragility	0.57%	0.44-0.60%
**4.EMA Flow Cytometry Analysis**		
Reduction of MFI in Erythrocyte E5'M	23.89%	<16%

Abbreviations：EMA：Eosin-5'-Maleimide, MFI:Mean Fluorescence Intensity.

Table 2. Summary of clinical phenotypes observed in the proband and family members

Relationship to the proband	Gender	Age	Clinical manifestations
Proband	Female	11	mild microcytic anemia, clinically apparent jaundice, mild splenomegaly
Mother	Female	36	mild microcytic anemia before splenectomy, normal hemoglobin level after splenectomy, no jaundice
Sister	Female	14	mild microcytic anemia, subclinical jaundice, mild splenomegaly
Father	Male	47	asymptomatic

Table 3. Hematological Parameters in Wild-Type and Ank1 c.5198G>A Knock-In Mice

Parameter	Wild-Type (n=24)	Mutant (n=49)	T-test p-value
RBC ($\times10^6/\mu L$)	10.41 ± 0.88	10.74 ± 1.02	0.065
HGB (g/L)	157.8 ± 9.5	159.7 ± 11.2	0.091
HCT (%)	52.1 ± 3.4	53.5 ± 3.9	0.078
MCV (fL)	50.9 ± 1.5	48.3 ± 1.8	0.002
MCH (pg)	15.2 ± 0.4	14.6 ± 0.5	0.015
MCHC (g/L)	302 ± 8	296 ± 10	0.038
RDW (%)	15.8 ± 1.2	15.5 ± 1.4	0.112
WBC ($\times10^3/\mu L$)	12.3 ± 3.2	13.1 ± 4.0	0.289
PLT ($\times10^3/\mu L$)	1032 ± 110	1067 ± 122	0.351

Table 4. Comparison of EMA Fluorescence Intensity Between Wild-Type and Ank1 c.5198G>A Knock-In Mice

Group (n)	Mean ± SD (%)	p-value (Mann-Whitney U Test)
Mutant (n=7)	-23.55 ± 7.84	$p < 0.01$
Wild type (n=4)	+10.00 ± 2.00	

Figure 1

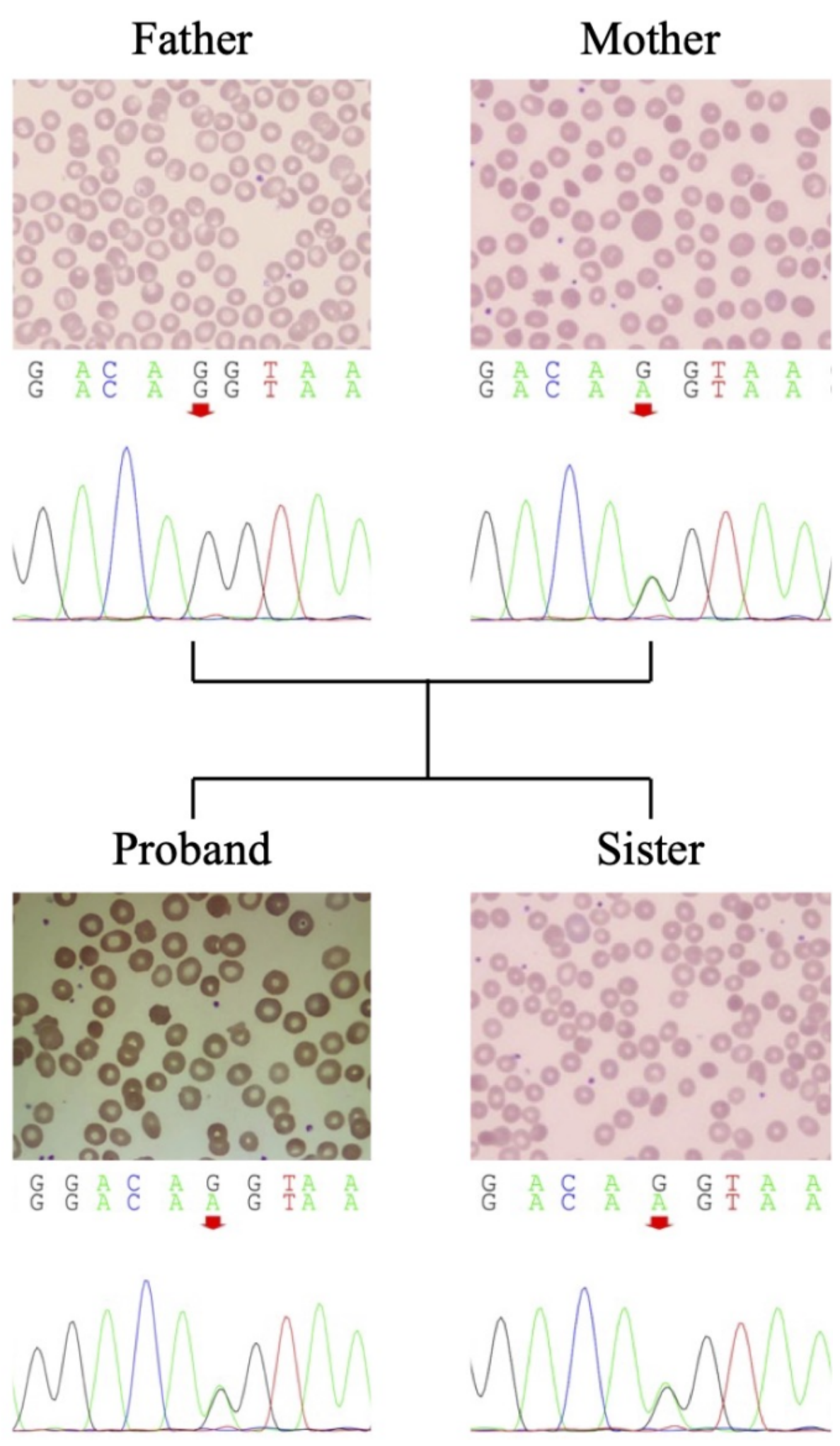

Figure1. Peripheral blood smears and Sanger sequencing of an HS-affected family carrying the Ank1 c.5096G>A (p.R1699K) mutation. Peripheral blood smears were shown in the top and Sanger sequencing confirms the Ank1 c.5096G>A (p.R1699K) mutation (red arrow) in below.

Figure 2

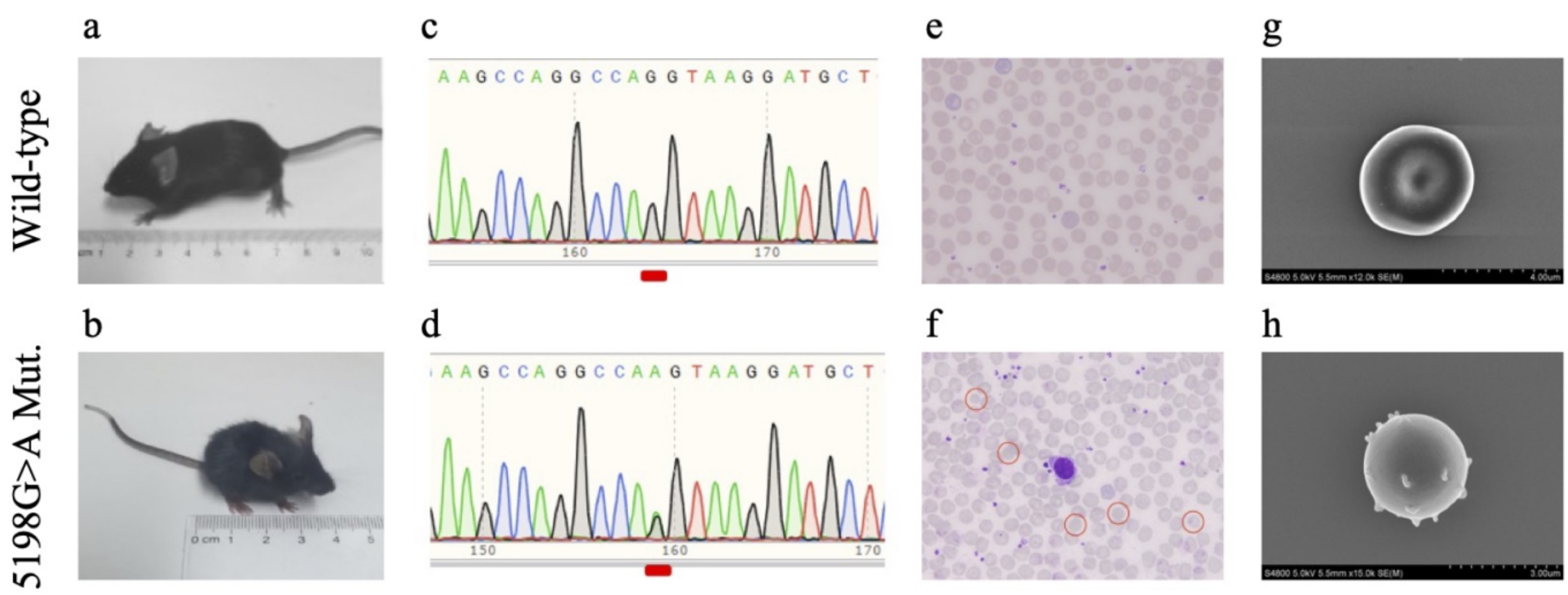

Figure 2. Hematological characterization of Ank1 c.5198G>A knock-in mice compared to wild-type mice. (a, b) Representative images of wild-type and Ank1 c.5198G>A knock-in mice. (c, d) Sanger sequencing images of wild-type and Ank1 c.5198G>A knock-in mice. (e, f) Peripheral blood smears (Wright-Giemsa staining). (g, h) Scanning electron microscopy (SEM) image.

Figure 3

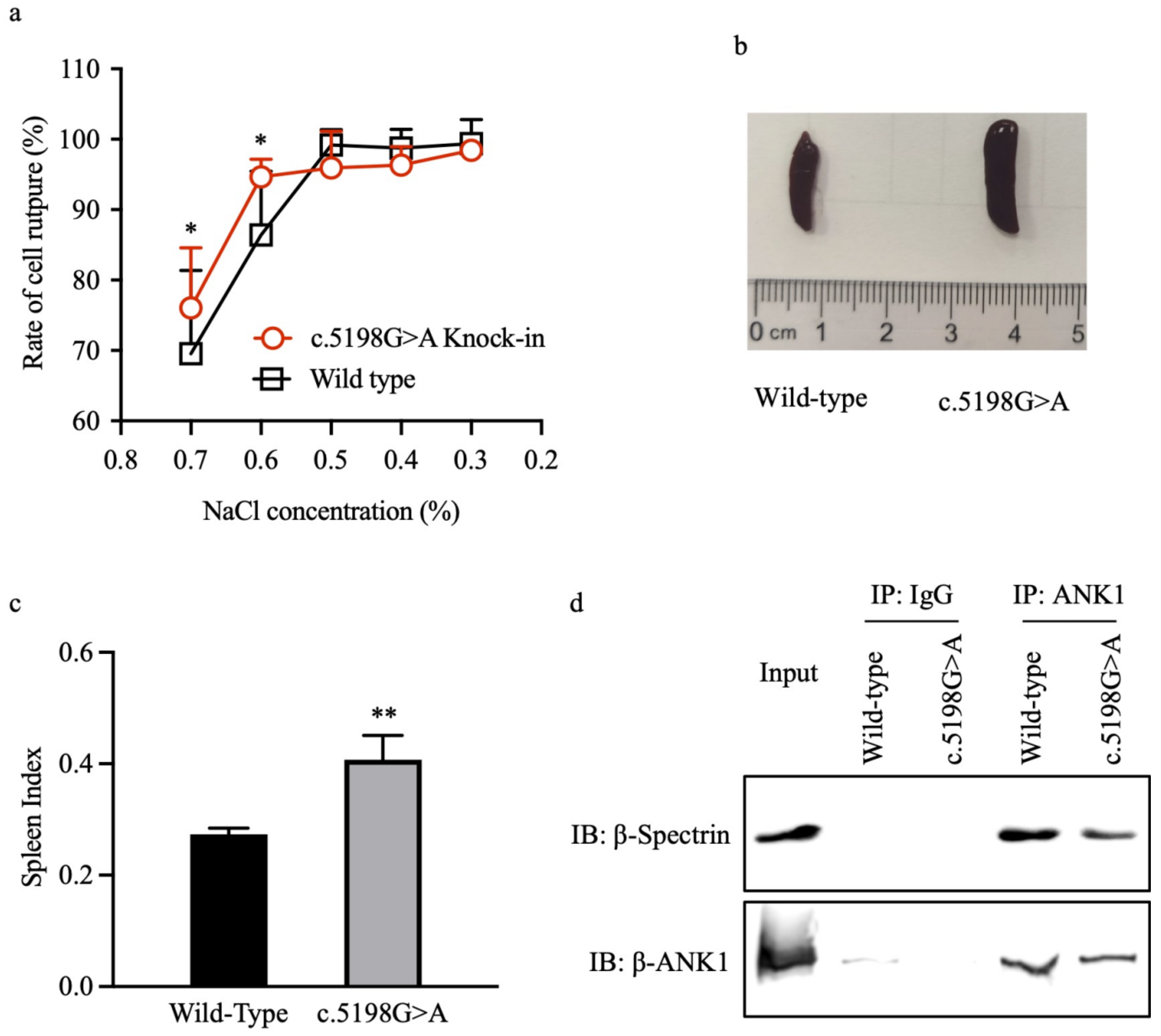

Figure 3. (a) Osmotic fragility assay, * $p < 0.05$ compared to wild type mice group. (b) Representative gross anatomical images of spleens from wild-type and Ank1 c.5198G>A knock-in mice. (c) Quantitative analysis of spleen index (spleen weight/body weight × 100%) in Ank1 c.5198G>A knock-in mice (n = 23) and wild-type mice (n = 23). ** $p < 0.01$) compared to wild type mice group. (d) Ank1 c.5198G>A (p.R1733K) mutation Reduced the association between Ankyrin and Spectrin. Co-immunoprecipitation (co-IP) assay, the pulling antibody was Ank1. Control immunoprecipitation was

performed using rabbit IgG. Protein samples from whole cell lysates were used to point at the position of the protein examined. IP, immunoprecipitated; IB, immunoblot.
